# Global and local identifiability analysis of a nonlinear biphasic constitutive model in confined compression

**DOI:** 10.1098/rsif.2024.0415

**Published:** 2024-11-13

**Authors:** John M. Peloquin, Dawn M. Elliott

**Affiliations:** ^1^ Department of Biomedical Engineering, University of Delaware, Newark, Delaware, US

**Keywords:** identifiability analysis, parameter estimation, soft tissue mechanics, cartilage, confined compression

## Abstract

Application of biomechanical models relies on model parameters estimated from experimental data. Parameter non-identifiability, when the same model output can be produced by many sets of parameter values, introduces severe errors yet has received relatively little attention in biomechanics and is subtle enough to remain unnoticed in the absence of deliberate verification. The present work develops a global identifiability analysis method in which cluster analysis and singular value decomposition are applied to vectors of parameter–output variable correlation coefficients. This method provides a visual representation of which specific experimental design elements are beneficial or harmful in terms of parameter identifiability, supporting the correction of deficiencies in the test protocol prior to testing physical specimens. The method was applied to a representative nonlinear biphasic model for cartilaginous tissue, demonstrating that confined compression data does not provide identifiability for the biphasic model parameters. This result was confirmed by two independent analyses: local analysis of the Hessian of a sum-of-squares error cost function and observation of the behaviour of two optimization algorithms. Therefore, confined compression data are insufficient for the calibration of general-purpose biphasic models. Identifiability analysis by these or other methods is strongly recommended when planning future experiments.

## 1. Introduction

Application of biomechanical models requires estimation of model parameters, to a reasonable error tolerance, from experimental data. Estimation of model parameters is subject to many sources of error [1–16]. *Non-identifiability* of parameters, when the same model output can be produced by many sets of parameter values, is particularly troublesome because it impacts every parameter estimate, cannot be corrected retrospectively, and is subtle enough to remain undetected in the absence of deliberate verification. Verification of parameter (non-)identifiability is called *identifiability analysis* [17–29]. Despite the importance of identifiability analysis, there are few prior examples of identifiability analysis methods being applied to tissue mechanics models [30–32]. The present work develops an identifiability analysis method well-suited to the study of tissue mechanics models, applies it to a practically significant example of a nonlinear biphasic constitutive model and test protocol, and compares its results with two established identifiability analysis methods.

**Figure 1.**
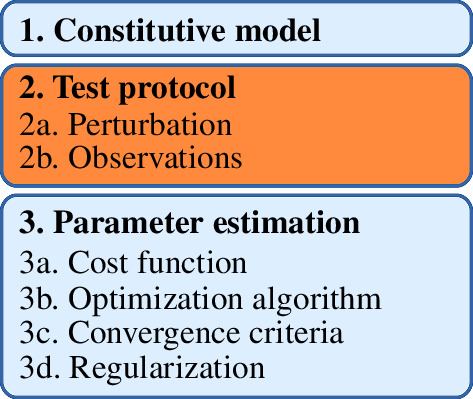
Development of a predictive tissue biomechanics model requires choosing (1) a constitutive model, (2) a test protocol to obtain experimental data, and (3) a method by which model parameters are estimated from the data. Overall success requires the success of each individual step. Identifiability analysis, the subject of the present paper, verifies that the choice of test protocol will allow estimation of the constitutive model’s parameters.

The development of a tissue biomechanics model has three major steps (figure 1), [7,14]. First, a constitutive model is chosen to define the properties of the tissue itself, e.g. the relationship between stress and strain in the tissue. The choice of constitutive model determines which physical processes will be represented in the overall model. Second, a test protocol is chosen and observations are collected from physical specimens. The test protocol defines the model input (the perturbations that would be applied to an experimental specimen) and output variables (the experimental observations). Third, an optimization algorithm is chosen to estimate parameter values. The optimizer adjusts the model parameters to minimize the difference between the experimental observations and the model’s predictions. If every possible set of parameter values corresponds to a unique set of model output values, point estimates of the true parameter values are obtainable and the parameters are identifiable; otherwise, they are not identifiable [19,33].

Identifiability is a mathematical property of the union of the constitutive model and the test protocol, and is independent of both experimental error and the choice of parameter estimation procedure. (If all potential model inputs were experimentally accessible, identifiability would instead be a property of the constitutive model alone.) In the parameter estimation stage, the effects of non-identifiability are easily misattributed to issues with the optimizer, but non-identifiability can only be solved by changing the model or the test protocol. The need to represent essential physical phenomena constrains the choice of model, and once data is collected, it is too late to change the test protocol. Therefore, to avoid wasting resources on experiments that are not fit for purpose, it is advisable to perform an *a priori* identifiability analysis immediately after choosing the test protocol.

Considering application to tissue mechanics, cartilaginous tissues such as the intervertebral disc, meniscus and knee articular cartilage are often modelled using nonlinear biphasic constitutive models [34–45]. Models of this type explain how a tissue’s interstitial fluid interacts with its solid matrix to produce emergent phenomena such as time dependence, fluid load support and lubrication [46–50]. Early biphasic models for tissue were linear and thus straightforward to analyse. It has long been known that confined compression allows estimation of aggregate modulus and fluid permeability, but not Poisson ratio, for a linear biphasic model [51–53]. Creep indentation, in contrast, allows the estimation of all three linear biphasic parameters [54–57]. These identifiability characteristics were discovered analytically.

**Figure 2.**
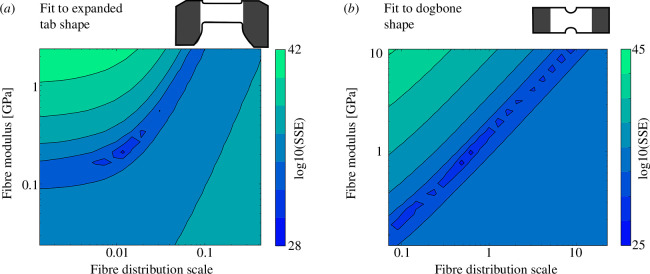
An example of parameter non-identifiability induced by specimen shape in a prior study. Bovine meniscus specimens were stretched uniaxially in the prevailing fibre direction. Some specimens were cut with expanded tabs and some were cut in a dogbone-like shape. The uniaxial tension data was fit with a three-parameter fibre recruitment model using the sum-of-squares error (SSE) as the fit metric. The SSE in a cross-section of parameter space is shown for two representative specimens: (*a*) The expanded tab specimen showed a fairly well-defined global minimum, indicating the model parameters were well-constrained and valid. (*b*) The dogbone specimen had a long valley of similar SSE values, indicating non-identifiability, which prevented identification of a unique set of model parameter values. Plotting cross-sections of the cost function in this manner to check identifiability becomes impractical as the number of parameters increases. Data from experiments reported in [60].

Nonlinear biphasic models are more easily analysed by numerical methods. Several numerical methods have been applied to check the identifiability of solid mechanics models. First, parameter estimation (optimization) can be repeated with several different initial guesses [58,59]. If all repetitions yield the same parameter estimate, the estimate is probably valid. (Its accuracy is a separate question.) If the estimates differ, there is no definite conclusion, as this outcome may be caused by problems other than non-identifiability. Second, the cost function in the neighbourhood of the parameter estimate can be examined visually (figure 2), in terms of its Hessian, or in terms of the model’s Fisher information matrix (FIM) [14,30,31,60,61]. If the cost function has a well-defined minimum (high curvature in all directions in parameter space), the parameters are identifiable; otherwise, they are not identifiable. The Hessian and FIM are related, under certain assumptions, to the parameters’ conditional posterior distributions [19]. Third, the posterior distributions can be estimated by Bayesian methods, with a large posterior variance indicating non-identifiability [32]. These methods are local; each analyses a single point in parameter space or data space at a time. They also aggregate the effects of all observations and thus cannot compare the impact on identifiability from different parts of the protocol, such as a loading ramp versus a stress relaxation hold. Although these methods are very useful, these characteristics are inconvenient when designing a test protocol prior to physical experimentation.

Identifiability analysis has been recognized as necessary due diligence in biomechanics model development but is not yet routine practice. With some notable exceptions [14,30,31], the problem tends to be approached from the perspective of *post hoc* troubleshooting of the optimization step rather than *a priori* verification during the experimental design phase. There is a need to develop and promote convenient identifiability analysis methods for tissue mechanics. The objective of this work was to develop and demonstrate an identifiability analysis method for tissue mechanics models with the following desirable characteristics:

— Operation on a parameter domain rather than a particular set of parameter values, since in an *a priori* analysis the true parameter values are not known. That is, it is desirable that the method be global, not local.
— Visualization of which parts of the constitutive model or protocol are responsible for (non-)identifiability to support decisions regarding simplification of the constitutive model (figure 1, step 1) or augmentation of the experimental protocol (figure 1, step 2).
— Interoperability with FEBio, a finite element software package commonly used for biomechanics simulations [62].
— Open source code.

A global identifiability analysis method meeting these characteristics is developed herein and applied to a nonlinear poroelastic constitutive model of cartilaginous tissue and a confined compression protocol as an example that is practically relevant to tissue biomechanics modelling. Two independent approaches, local identifiability analysis of the cost function and observation of optimizer behaviour on synthetic data, are used to verify the results.

## 2. Methods

### 2.1. Constitutive model

Cartilaginous soft tissue tested in confined compression was chosen as a concrete, practically significant example for identifiability analysis. The choice to begin with confined compression was somewhat arbitrary; identifiability analysis is warranted for all test methods that are used to fit constitutive models [11,63–66]. The constitutive model was chosen to represent the existing literature while still including all physical phenomena known to be key aspects of cartilaginous soft tissue behaviour in confined compression. That is, the analysis’ constitutive model had to exhibit nonlinear elasticity, strain-dependent permeability and osmotic pressure [16,35,67–69]. Anisotropy was not included because confined compression prevents the development of transverse tensile strain and thus minimizes the effects of anisotropy [35,70–72]. To satisfy these requirements, a biphasic (poroelastic) mixture consisting of a Holmes–Mow elastic solid with Holmes–Mow strain-dependent permeability and Donnan equilibrium osmotic swelling pressure was chosen as the constitutive model [34–36].

The strain energy $\Psi_{HM}$ from the Holmes–Mow solid is a function of the first and second invariants of the right Cauchy–Green tensor $I_{1}$ and $I_{2}$ and the volume ratio J. Its parameters are elastic modulus E, Poisson ratio ν and nonlinearity β.


$$\begin{array}{c} \Psi_{HM}\left(I_{1},I_{2},J\right)=\frac{E\left(1-\nu \right)}{4\beta \left(1+\nu \right)\left(1-2\nu \right)}\left\{exp\left[\beta \left(\frac{3\nu -1}{\nu -1}\left(I_{1}-3\right)+\frac{\nu }{1-\nu }\left(I_{2}-3\right)-{\left(lnJ\right)}^{2}\right)\right]-1\right\} \end{array}.$$

The Holmes–Mow strain-dependent permeability k is a function of J, with four parameters: strain-free permeability $k_{0}$, exponential coefficient M, power law exponent α and strain-free porosity $\phi_{w}^{0}$. Note that many so-called porosity values reported in the literature appear to be mass fractions rather than volume fractions; a valid measurement is provided by [73].


$$\begin{array}{c} k\left(J\right)=k_{0}{\left(\frac{J-1+\phi_{w}^{0}}{\phi_{w}^{0}}\right)}^{\alpha }exp\left(\frac{M}{2}\left(J^{2}-1\right)\right) \end{array}.$$

The Donnan equilibrium swelling pressure p is a function of temperature T, the osmolarity of the external solution $c_{*}$ and J. It has the parameters osmotic coefficient Φ, strain-free fixed charge density ${\text{FCD}}_{0}$ and strain-free porosity $\phi_{w}^{0}$. Here, T was set to 294 K (room temperature) and $c_{*}$ was set to 300 mOsm (standard saline).


$$p\left(T,\;c_{*},J\right)=\Phi RT\left(\sqrt{{\left(FCD\right)}^{2}+{\left(c_{*}\right)}^{2}}-c_{*}\right),$$

$$\text{FCD}\left(J\right)=\frac{\phi_{w}^{0}}{J-1+\phi_{w}^{0}}FCD_{0}.$$

The total stress was the sum of all constituents, following standard biphasic mixture theory [74,75]. In total, the model had 10 parameters (table 1).

**Table 1.** Parameter ranges used in identifiability analysis.

parameter	min	max	relevant references
Elastic modulus, E	130 kPa	470 kPa	[35,71]
Poisson ratio, ν	0	0.3	[35,71]
Elastic nonlinearity, β	0.04	0.5	[35,71]
Strain-free permeability, $k_{0}$	1 × 10^−4^ mm^4^ (Ns)^−1^	20 × 10^−4^ mm^4^ (Ns)^−1^	[35,71]
Exponential permeability coefficient, M	1.7	5.8	[35,71]
Power law permeability exponent, α	1	2	[35,36,71]
Strain-free porosity, $\phi_{w}^{0}$	0.6	0.87	[73,76]
Strain-free fixed charge density, ${\text{FCD}}_{0}$	120 mM	315 mM	[35]
Osmotic coefficient, Φ	0.9	1.0	[35,36,71]
Initial swelling fraction, $f_{0}$	0.0	0.5	[35,77]

### 2.2. Simulated test protocol

**Figure 3.**
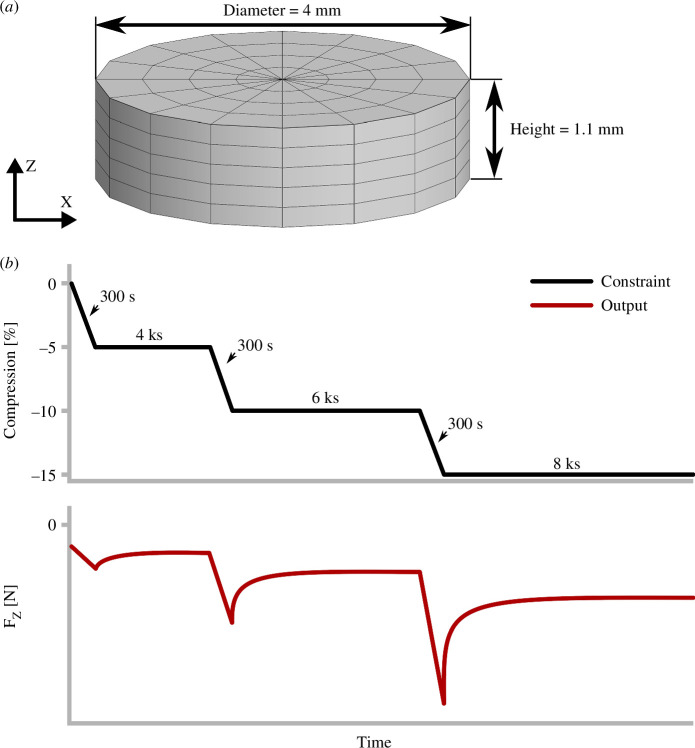
(*a*) Specimen dimensions and mesh in its initial strain state ($f_{0}\varepsilon_{fs}$), experimentally defined as 0% compression. The bottom surface was free-draining. (*b*) Schematic of the applied incremental stress relaxation confined compression protocol and resulting reaction force.

The simulated test protocol represented a typical confined compression stress relaxation experiment in which the specimen is equilibrated with a 0.15 M saline solution, with swelling restricted to its initial (as-prepared, when cut to shape) height and diameter, and then compressed by three increments of 5% compression calculated relative to this initial height (figure 3) [35,71]. This initial strain originates from *in situ* (*in vivo*) osmotic swelling; it is non-zero and unknown. This state of initial strain has been referred to as prestrain, prestress and residual stress [36,77–79]. The experimental initial state is, therefore, not consistent with the zero-strain reference state used in the constitutive equations, and it is necessary to account for this discrepancy in the finite element analysis (FEA) simulation [35,77,78,80,81].

Direct manipulation of initial strain in biphasic FEA simulations is non-trivial, so the initial strain in this study was assumed to be isotropic and was indirectly parametrized by the ‘initial swelling fraction’ $f_{0}$, defined as follows. Given a set of values for E, ν, β, $\phi_{w}^{0}$, ${\text{FCD}}_{0}$ and Φ, the free swelling engineering strain at equilibrium is $\varepsilon_{fs}$. The initial engineering strain in the FEA simulation was accordingly set to $f_{0}\varepsilon_{fs}$, $f_{0}\in \left[0,1\right]$, to cover the plausible range of *in situ* swelling strain. To produce the initial dimensions as specified in figure 3*a* with the initial strain present, the FEA mesh dimensions were scaled by a factor of ${\left(1+f_{0}\varepsilon_{fs}\right)}^{-1}$. Each FEA simulation began by deforming the mesh to its initial dimensions and allowing fluid equilibration, producing the desired initial diameter = 4 mm, initial height = 1.1 mm and initial strain = $f_{0}\varepsilon_{fs}$. The 5% compression increments were calculated relative to this 1.1 mm initial height and applied along the z-axis. The output variable used in the identifiability analysis was the reaction z-force, F_z_.

### 2.3. Finite element model

The confined compression test was simulated using the finite element software FEBio [62]. The FEBio XML files that defined the model were generated using the Python library waffleiron (https://github.com/jpeloquin/waffleiron). The mesh consisted of a cylinder of 8-node hexahedral linear elements with a core of 6-node pentahedral linear elements at the centre, with NE_C_ = 16 elements along the circumference, NE_R_ = 4 along the radius and NE_Z_ = 5 along the height (figure 3*a*). Run times were acceptable (approx. 10 s on one core) despite not exploiting symmetry. To confirm that the number of elements contributed negligible variance compared to the material parameters, the global identifiability analysis procedure described below was repeated as a mesh sensitivity analysis using NE_C_, NE_R_ and NE_Z_ as the varied parameters, with the results given in electronic supplementary material. The bottom nodes were set to be free-draining and had fixed position in z. After the initial strain was applied according to $f_{0}$, the side nodes were fixed in x and y and the top nodes’ z-displacements were constrained via tied contact to follow the confined compression incremental stress relaxation protocol (figure 3*b*). Nine time points were used per ramp segment and 15 time points, logarithmically spaced in time, per hold segment.

### 2.4. Identifiability analysis

Three analyses were performed: (i) a global identifiability analysis based on correlation coefficients, (ii) a local identifiability analysis based on the curvature of a sum of squares error (SSE) function as used in parameter estimation, and (iii) an examination of optimizer behaviour during parameter estimation. The global analysis method is a new variation on existing identifiability analysis concepts, specialized to guide the design of tissue biomechanics experiments. The other two methods have been used in multiple fields. All three analyses involve sampling the parameter space Θ formed by the permissible potential values of each parameter. Here, these ranges were chosen using experimental data for the intervertebral disc cartilaginous endplate to reflect the parameters’ range of plausible true values, including both natural variation and uncertainty from lack of information (table 1). For parameters with reported mean and s.d., the range was set to the [0.159, 0.841] quantile interval for the corresponding lognormal distribution. The Python software that ran these analyses was published here for the first time (https://github.com/jpeloquin/spamneggs).

#### 2.4.1. Global identifiability analysis

The global identifiability analysis used full factorial sampling with three equally spaced levels per parameter to fully span the parameter space (table 1), producing 59 049 samples. The test protocol was simulated for each sample using the finite element model described above, and for each time point the Pearson correlation coefficient was calculated between each parameter and F_z_. The vector of correlation coefficients for a parameter, or ‘correlation vector’, measures the parameter’s direct effect on F_z_ across time. Differences between the parameters’ correlation vectors were assessed using hierarchical cluster analysis. Specifically, clusters were assigned using the unweighted pair group method with an arithmetic mean (UPGMA) algorithm with unsigned cosine distance as the pairwise distance metric. Since this cluster analysis only assessed pairwise differences, whereas the effects of one parameter might be mimicked by others in combination, linear independence of parameter effects was additionally assessed using singular value decomposition (SVD) of the matrix of correlation vectors. Due to the large number of samples, several automatic error checks were implemented to verify that each simulation actually followed the specified test protocol. Out of 59 049 simulations, 295 deviated from the protocol or exhibited other errors and were excluded (see electronic supplementary material).

#### 2.4.2. Local identifiability analysis

The local identifiability analysis analysed the curvature (Hessian matrix) of the SSE cost function at the true parameter values $\theta^{*}$ for 10 random parameter samples, treating the parameter ranges as independent uniform distributions [31,32,82–84]. The FEA model is considered as $f\left(\theta \right)=F_{z}$; θ∈Θ, with Θ defined as the parameter space covering the ranges in table 1. Then, for each sample (set of true parameter values) $\theta^{*}\in \Theta$, the cost function $j\left(\theta ;\theta^{*}\right)=\sum_{i}{\left(f{\left(\theta \right)}_{i}-f{\left(\theta^{*}\right)}_{i}\right)}^{2}$ and its Hessian $H\left(\theta ;\theta^{*}\right)=\frac{\partial^{2}j\left(\theta ;\theta^{*}\right)}{\partial \theta_{i}\partial \theta_{j}}$. To avoid loss of precision the cost function was implemented using the Shewchuck summation algorithm [85]. The Hessian was calculated at $\theta^{*}$ using second-order finite differences with a fixed step size, with 201 function evaluations (i.e. FEA simulations) per Hessian evaluation. To address the dissimilarity of units and thus a lack of common scale between parameters, the parameters were scaled to [0, 1], with 0 = the range’s minimum and 1 = the range’s maximum [86]. The step size in scaled parameter units was 0.01, which was selected from a step size sweep between 10^−8^ and 10^−2^. $H\left(\theta^{*}\right)$ measures the curvature of the cost function at the true parameter values, and thus the best-case degree of constraint on the parameters. From an identifiability perspective, the outcomes of interest are the Hessian’s eigenvalues.

#### 2.4.3. Optimizer behaviour

To test if non-identifiability detected in the analyses described above translated into observable parameter estimation issues, optimizer behaviour was examined for 10 sets of parameter values $\theta^{*}$ obtained by uniform random sampling of the parameter ranges (table 1) and two optimization algorithms. For each sample $\theta^{*}$, a parameter estimate $\hat{\theta }$ was obtained by minimization of the SSE cost function used in the local identifiability analysis using, separately, the Nelder–Mead and trust-region constrained algorithms provided by scipy.optimize.minimize. The use of both a direct search procedure and a quasi-Newton method covers two of the major classes of optimizers relevant to this estimation problem, the third being Marquardt’s method or a variant thereof (not examined here) [87]. Each optimization was initialized using a point in Θ randomly selected in the same manner as $\theta^{*}$ (but not equal to $\theta^{*}$). The parameters were scaled to [0, 1] as in the local identifiability analysis for the same reasons. The optimizer’s state at each iteration, including the current parameter estimate, $F_{z}$ output, cost function value and the cost function gradient (only for the trust-region constrained algorithm) was recorded using Zarr (https://github.com/zarr-developers/zarr-python) for subsequent review. The main outcome of interest is the parameter estimation error, $\hat{\theta }-\theta^{*}$.

## 3. Results

### 3.1. Global identifiability analysis

**Figure 4.**
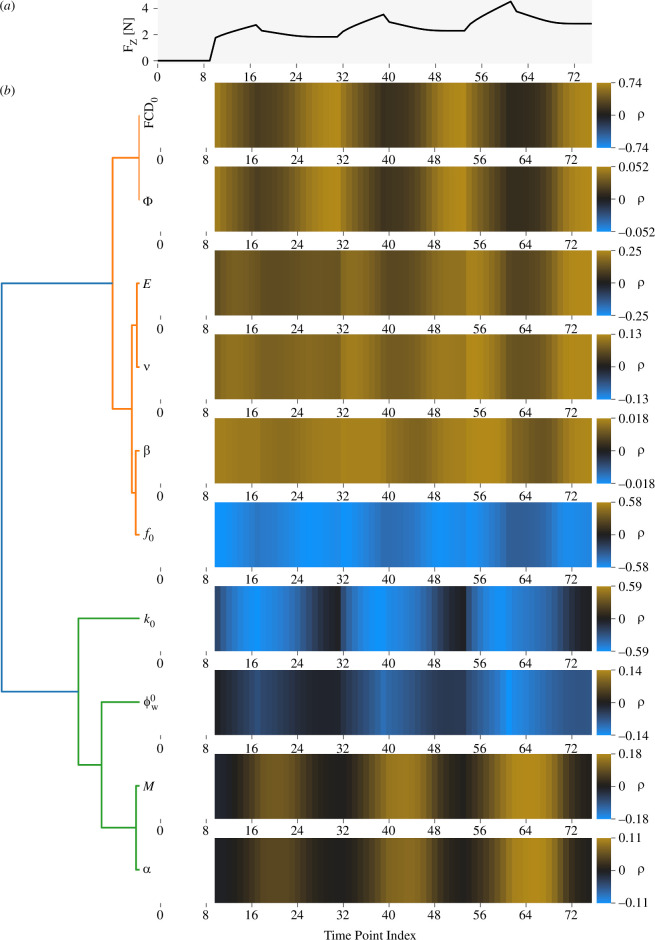
Global identifiability analysis Pearson correlation coefficients, ρ, between each parameter and the reaction force at each time point across all samples drawn from parameter space. (*a*) Model output from one parameter sample to indicate the part of the test protocol to which each time point belongs. (*b*) Correlation vectors, consisting of one correlation coefficient per time point, between each parameter’s sampled values and F_z_ at that time point. Each horizontal heatmap displays one correlation vector. Parameters that affect the output similarly are clustered together, and parameters that are tightly clustered are probably non-identifiable.

Each parameter’s direct effects across time were quantified as a vector of the correlation coefficients, for each time point, between the parameter’s sampled values and the model output F_z_ (figure 4). These correlation vectors form the basis of the global identifiability analysis. Some parameters had dissimilar correlation vectors and thus unique effects on F_z_, whereas others had very similar correlation vectors and thus redundant effects. For example, FCD_0_ and $k_{0}$ had effects that were very different from each other. FCD_0_ had greatest influence over the reaction forces at the end of each hold, whereas $k_{0}$ had the greatest influence over the peak reaction forces at the end of each ramp. There were four pairs of parameters with very similar effects: (i) FCD_0_ and Φ, (ii) E and ν, (iii) and β and $f_{0}$, and (iv) M and α. The parameters in each near-identical pair have degenerate effects and are thus non-identifiable. The remaining parameters had intermediate distinctiveness, forming five−six distinct clusters.

The correlation vectors, in addition to their use for identifiability analysis, can also be interpreted as a global sensitivity analysis (figure 4, referring to correlation magnitudes). The reaction force F_z_ was most strongly correlated to $k_{0}$, FCD_0_ and initial swelling fraction $f_{0}$. β and Φ had negligible effects on the output and are therefore individually non-identifiable for that reason alone. As the correlation vectors only capture direct effects, first-order and total sensitivity indices were also calculated (electronic supplementary material, figure S1). There were substantial differences between first-order and total indices for $k_{0}$, M, α, $\phi_{w}^{0}$, FCD_0_ and $f_{0}$, revealing that these parameters had substantial indirect effects. Analysis of this model and test protocol should, therefore, use a sampling strategy that covers the parameter space and includes parameter interactions, which the global analysis here does.

**Figure 5.**
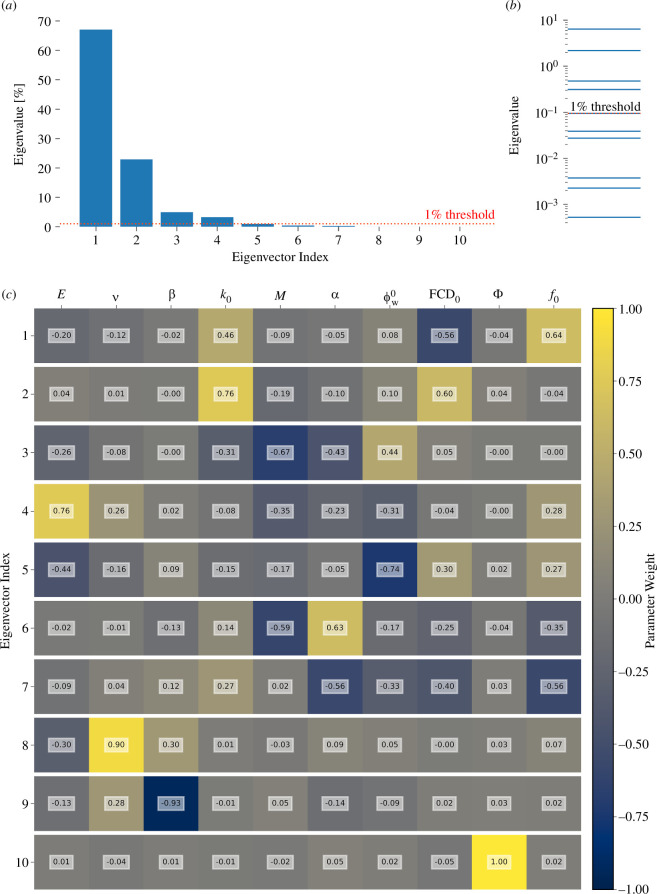
Global identifiability analysis eigenvalues and eigenvectors from SVD of the matrix of parameter–output correlation vectors shown in figure 4. (*a*) Eigenvalues normalized to sum to 100%. (*b*) Eigenvalues on a log scale. In (*a*) and (*b*), small eigenvalues indicate non-identifiability in the direction of their associated eigenvectors. The red dotted line indicates an identifiability threshold equal to 1% of the sum of all eigenvalues. (*c*) Parameter weights that comprise each eigenvector.

The above results show that some parameters are non-identifiable, but the problem may be even worse than apparent so far because a seemingly distinct parameter effect might be mimicked by varying other parameters in combination [88]. This possibility was addressed using SVD of the matrix of correlation vectors shown in figure 4. Each resulting eigenvector is a direction in parameter space, and its eigenvalue indicates the variance in the parameters’ correlation vectors associated with that direction. Eigenvalues that are small, therefore, indicate non-identifiability in the direction of the associated eigenvector. The eigenvalues are spread across five orders of magnitude, with only four eigenvalues greater than 1% of the total (figure 5*a,b*). The three least important eigenvectors are mainly weighted towards the original ν, β and Φ basis vectors (figure 5*c*). The more important eigenvectors are weighted towards multiple parameters. Overall, the eigenanalysis indicates that the confined compression protocol effectively constrains only four−five directions (degrees of freedom) in parameter space. Parameters can be varied in combination along the other directions with minimal effect on the model output.

### 3.2. Local identifiability analysis

**Figure 6.**
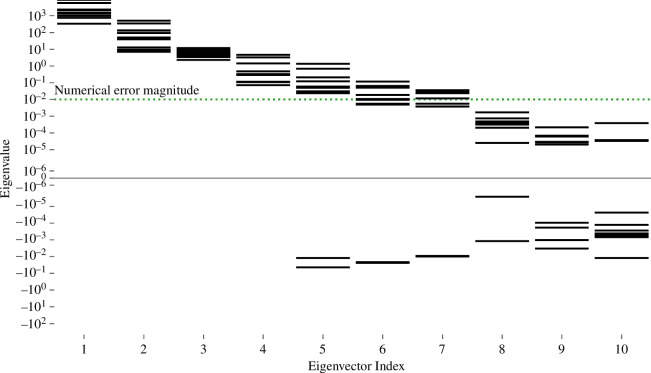
Local identifiability analysis. Eigenvalues of the Hessian of the SSE cost function evaluated at the true parameter values for 10 parameter samples. The green dotted line marks the estimated magnitude of the numerical error in the eigenvalues. Small eigenvalues indicate non-identifiability along the corresponding eigenvectors in parameter space.

To confirm the results of the global identifiability analysis, local identifiability analysis was performed for 10 samples from parameter space using the Hessian (i.e. curvature) of the SSE function evaluated at the true parameter values. In each sample, the eigenvalues of the Hessian varied across approximately nine orders of magnitude (figure 6). Critically, eigenvalues for eigenvectors 5−10 were sometimes negative. A mixture of negative and positive eigenvalues indicates a saddle point, which at the true parameter values can only be due to uncertainty in the output variables. Here, the only source of uncertainty is numerical error.

The amount of numerical error was estimated by systematically varying the finite difference step size used to calculate the Hessian and observing its resulting variation, as described in the methods section. This step size sweep indicated that the Hessian had numerical error of approximately 10^−2^ at the optimal step size, comparable to that previously observed in another FEBio-based analysis [31]. The step size sweeps often showed no truncation error regime, suggesting condition error was dominant [89,90]. When a truncation error regime was observed, the transition from condition error to truncation error occurred at step sizes between 10^−4^ and 10^−2^. By the Bauer–Fike theorem, this error will cause error of similar magnitude in the eigenvalues, and negative eigenvalues indeed start to be observed when the eigenvalues’ absolute values approach approximately 10^−2^ (figure 6, green dotted line). Overall, only four eigenvectors consistently have eigenvectors above the numerical error threshold; therefore, the confined compression protocol effectively constrained only four directions (degrees of freedom) in parameter space.

### 3.3. Optimizer behaviour

**Figure 7.**
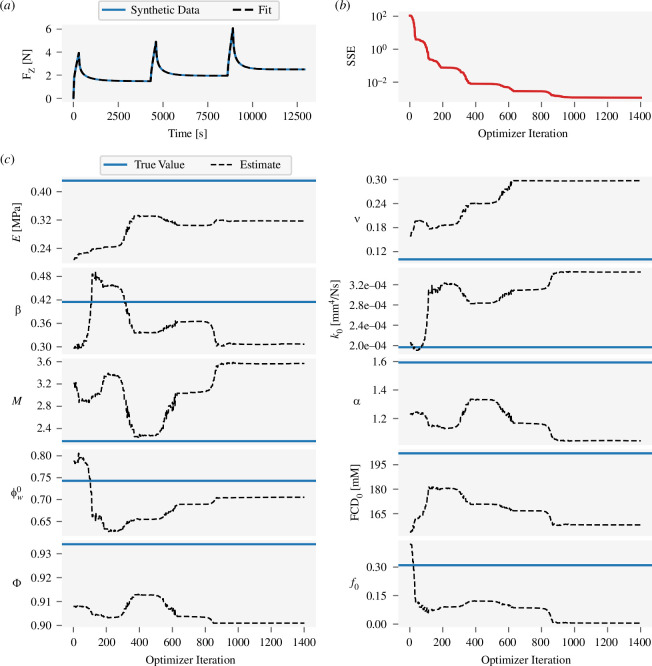
Nelder–Mead optimization behaviour for a representative ‘ground truth’ sample $\theta^{*}$ and synthetic data F_z_($\theta^{*}$). (*a*) Fit at the final, converged, iteration. (*b*) Evolution of the cost function across iterations. (*c*) Evolution of the parameter estimates across iterations. The final fit is qualitatively indistinguishable from the ground truth synthetic data, but the parameter estimates have substantial error, which indicates non-identifiability.

**Figure 8.**
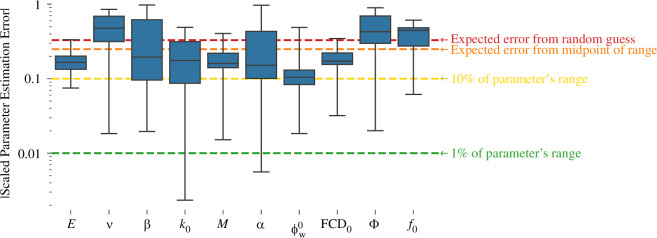
Parameter estimation errors, in scaled parameter space, from applying Nelder–Mead optimization to synthetic data (*n* = 10). Key error thresholds are indicated with dotted lines, referring to the parameter ranges in table 1. The ‘random guess’ is a random uniform sample. The box plot shows the median, quartiles and minimum/maximum.

To clearly demonstrate that the identifiability issues noted above impact parameter estimation, the performance of two optimization algorithms, Nelder–Mead and trust-region constrained optimization, was examined for synthetic error-free (‘ground truth’) data generated from 10 random samples $\theta^{*}$ from parameter space. The optimizer estimate is $\hat{\theta }$ and the estimation error is $\hat{\theta }-\theta^{*}$. For each sample, the Nelder–Mead algorithm produced an excellent fit to the data, with model output, F_z_, indistinguishable from the ground truth (figure 7*a*,*b*). Nonetheless, the estimation error was substantial, and the estimates often moved away from the true values as the SSE improved (figure 7*b*,*c*). Across all 10 samples, the median error for three parameters, ν, Φ and $f_{0}$, was greater than would be expected from simply drawing a random uniform sample from their allowed range (figure 8); i.e. the optimization-derived estimates were worse than guessing at random. Median error for all parameter estimates from the Nelder–Mead algorithm exceeded 10% of the parameter’s allowed range.

The trust-region constrained algorithm performed worse than the Nelder–Mead algorithm, producing fits that noticeably differed from the ground truth value, with similar estimation errors. Since the trust-region constrained results do not provide any additional insight regarding identifiability, they are not discussed further. Animations of the progress of each optimization are provided in the electronic supplementary material. The observation of large estimation errors for all parameters combined with good fits (small SSE) is the hallmark of non-identifiability, confirming the results of the previous two analyses.

## 4. Discussion

### 4.1. Summary

This work developed and demonstrated a global identifiability analysis method for tissue mechanics models. This global identifiability analysis method, local identifiability analysis and observation of optimizer behaviour all indicated that data from an incremental confined compression stress relaxation test protocol only constrains 4−5 out of 10 degrees of freedom in the parameter space of a nonlinear biphasic constitutive model. The parameters may take on many different values with negligible change in the model output and are, therefore, non-identifiable. Since two parameter estimates that produce the same output with one geometry and loading condition are unlikely to do the same across all other geometries and loading conditions, a model using parameter estimates obtained under conditions of non-identifiability must be considered miscalibrated for most purposes [25]. There can be no expectation that such a model will generalize to other contexts, provide mechanistic insight or support experimental hypothesis testing by comparison of parameter estimates. Nonlinear biphasic models calibrated using confined compression data are therefore suspect, and continued development of models for cartilaginous tissue must not rely solely on confined compression data. The code to run these identifiability analyses is provided in this article’s electronic supplementary material so they can be applied to other constitutive models and test protocols.

### 4.2. Fixing non-identifiability

Non-identifiability is an experimental design flaw, but whether the flaw lies in the choice of constitutive model or the choice of test protocol is a matter of perspective. If the flaw is said to lie with the test protocol, the perspective is that the protocol provides too little information (for the chosen constitutive model) and needs to be augmented to provide information sufficient to constrain every model parameter. Designing a valid test protocol is easier with an accessible, complete view of the parameters’ effects. The global identifiability analysis presented here combines elements from analysis of high-dimensional genomics data with existing identifiability analysis methods [19,23,91,92], showing the effects of each parameter for every time point and output variable. This visual representation clarifies which specific design elements are beneficial or harmful in terms of identifiability, providing useful feedback on the experimental design.

If the flaw is said to lie with the constitutive model, the perspective is that the model is too complex (for the chosen test protocol) and needs to be simplified to have fewer degrees of freedom and, thus, parameters. Model simplification, also called model reduction, is commonly recommended to resolve parameter estimation issues. It can be applied after data collection is complete, but altering the model alters the research question being asked and may compromise the work’s scientific merit [93,94]. In tissue biomechanics, the choice of constitutive model is often constrained by the need to include the tissue’s essential physical phenomena, so the simplest satisfactory model is often chosen *a priori*. For example, altering the model presented here to use constant permeability would remove three parameters (ϕ, α and M), but this would contradict evidence that permeability is strain-dependent in hydrogels and hydrated soft tissues [52,57,67,95,96]. Nor does simplification by removal of physical behaviours necessarily improve identifiability. For example, elastic Hertzian indentation only allows identification of the reduced modulus, but biphasic Hertzian indentation allows identification of both Young’s modulus and Poisson’s ratio [54–57,97]. The tissue’s physical characteristics matter, and it is better to refine the protocol to match the tissue.

### 4.3. Alternative experimental protocols

Other experimental protocols are likely to perform better than confined compression, which (except for the initial swelling) is essentially a one-dimensional test. In the context of linear biphasic models, indentation allows estimation of E, ν and $k_{0}$ [54–57]. Indentation has been extended to also measure either fixed charge density [98] or tension–compression nonlinearity [44,99], again for linear models. Unconfined compression is also effective for linear models [68,100–102].

Nonlinear models may pose greater challenges. Hessian-based local identifiability analysis has shown that several two-parameter nonlinear elastic models for soft tissue were non-identifiable using indentation reaction force alone, and that identifiability was restored by including full-field surface displacements [31]. However, including poroelasticity in the model may improve identifiability of nonlinear models from indentation data in the same way it does for linear models. Very few models and experimental protocols used for hydrated soft tissue have been subjected to identifiability analysis, and there are many of both, so considerable further work is required in this area. Automated identifiability analysis, such as with the software provided here (https://github.com/jpeloquin/spamneggs), will help by facilitating use of identifiability analysis during routine experimental planning.

### 4.4. Limitations and inconveniences

Ideally, an identifiability analysis would offer a guarantee that the model is either identifiable or not. Unfortunately, the analysis requires some subjective choices that may influence the results. First, the choice of parameter domain will influence the relative importance of parameters and may exclude regions of parameter space with unique parameter effects, if the model and protocol are capable of producing such regions. Therefore, there is a natural tendency to try to set the ranges using data. However, an identifiability analysis is most needed when parameter estimates are unavailable or not known to be trustworthy. For example, the present analysis invalidated the literature parameter estimates used to define its parameter ranges (table 1). Retrospective analysis is also not the normal application of identifiability analysis, even though it has become necessary due to the absence of identifiability analysis in prior work. In prospective experimental planning, there will typically be no prior parameter estimates, trustworthy or otherwise, and the parameter ranges must be set by educated guesswork. The goal is to set the parameter ranges to as broad a range as possible without producing unrealistic or implausible values in the simulated output variables, in the context of the specific model and protocol under consideration. The ranges do not need to be exact. Any global analysis explores a much greater swathe of parameter space than a local analysis, and local analyses are still useful. Experimental design is iterative. If identifiability analysis reveals a deficiency in the experimental design, adjustment of the parameter ranges is unhelpful; instead, the model or test protocol must change. If identifiability analysis does not reveal a problem, it is then worthwhile to acquire pilot data and attempt parameter estimation (figure 1, step 3). Whether the identifiability analysis needs to be revisited would then depend on the outcome of the pilot experiments.

Second, the results are on a continuous scale; a binary transition between identifiability and non-identifiability is imposed during interpretation of the results. The choice of identifiability threshold nonetheless has limited impact in practice. The eigenvalues in both the global and local analyses span multiple orders of magnitude. Most eigenvalues are far from any reasonable choice of identifiability threshold, and borderline identifiability is still undesirable because it will probably compound with other sources of experimental error. Third, identifiability analysis assumes that the choice of constitutive model and the representation of the test protocol in the simulation is consistent with the physical system, which is never completely true. Fourth, some identifiability analysis methods, albeit not the global analysis presented here, are also specific to the chosen optimization procedure. Overall, identifiability analysis is similar to an *a priori* power analysis in that it can show the strength or weakness of a study’s design, but it cannot guarantee success or failure. It is also similar to a power analysis in that it may need to be revisited as the available information changes, which makes automation essential.

### 4.5. Alternative approaches to identifiability analysis

In addition to dedicated identifiability analysis methods, inverse uncertainty analysis can also provide information about parameter identifiability [4,32,103–106]. Specifically, given the model $f\left(\theta \right)=y$, any method that computes the probability distribution of θ conditional on y should produce large (effectively infinite) confidence intervals for any non-identifiable components of θ. Methods that attempt to construct the inverse model $f^{-1}(y)=\theta$ may also be useful in that they should fail if the model is non-identifiable. However, if an inverse method can fail for reasons unrelated to identifiability, it is not particularly useful for identifiability analysis. Forward uncertainty analysis, which computes uncertainties in y given a probability distribution for θ, is also not relevant to identifiability even though it is crucial when applying models to practical problems [105,107–109].

Asymptotic confidence intervals may also be constructed from the inverse of the Hessian, subject to certain assumptions [19,87,110,111]. Results from a Hessian-based local identifiability analysis, like the one shown here, can, therefore, also be presented as an uncertainty analysis [84]. A non-identifiable model’s Hessian is, of course, non-invertible, but this difficulty may be overcome by construction of a surrogate pseudovariance matrix by generalized Cholesky decomposition [93].

Note that when a method that produces a posterior distribution is used as an identifiability analysis method, the posterior distribution is *not* an estimate of uncertainty, as measurement error is absent. The result is better viewed as reflecting the information gain from experimental observations. The threshold between identifiability and non-identifiability, therefore, remains somewhat subjective in the same way as discussed above. There is considerable room for future work to develop theoretical foundations for interpretation and potential equivalence of the various identifiability-related metrics. The suitability of various identifiability analysis methods for biomechanical models will also become clearer with use. All three identifiability analysis methods examined here supported the same conclusion, so it is possible that the choice of method does not matter much, and any identifiability analysis is better than none.

## 5. Conclusion

A global identifiability analysis method, in which cluster analysis and singular value decomposition were applied to vectors of parameter–output variable correlation coefficients, was introduced and applied to a representative nonlinear biphasic model for cartilaginous tissue. Confined compression incremental stress relaxation was shown to not provide parameter identifiability for this model, meaning that many combinations of parameter values can produce the same reaction force output. In other words, the parameter estimation problem is ‘ill-posed’ or ‘underdetermined’. This conclusion was confirmed by two independent analyses using different techniques: local analysis of the Hessian of a SSE cost function, and observation of the behaviour of two optimization algorithms. Data from confined compression tests has historically been an important source of parameter estimates for nonlinear biphasic models. These results indicate confined compression is insufficient for general-purpose calibration of these models. *A priori* identifiability analysis, by these or other methods, is strongly recommended for future work intended to support the modelling of tissue mechanics.

## Data Availability

All source code written by the authors and used in the preparation of this paper is included in the electronic supplementary material in a zip file. This file provides an archival snapshot of the code's state at the time of manuscript preparation. The docx file in the electronic supplementary material provides a description of the contents of each script and package. Supplementary material is available online [112].

## References

[1] Fung YC. 1993 Biomechanics: mechanical properties of living tissues. New York, NY: Springer.

[2] Holzapfel GA, Ogden RW. 2009 On planar biaxial tests for anisotropic nonlinearly elastic solids: a continuum mechanical framework. Math. Mech. Solids **14**, 474–489. (10.1177/1081286507084411)

[3] Safa BN, Bloom ET, Lee AH, Santare MH, Elliott DM. 2020 Evaluation of transverse poroelastic mechanics of tendon using osmotic loading and biphasic mixture finite element modeling. J. Biomech. **109**, 109892. (10.1016/j.jbiomech.2020.109892) 32807341 PMC7438606

[4] Jakeman JD. 2022 PyApprox: enabling efficient model analysis. U.S. Department of Energy, Office of Scientific and Technical Information. (10.2172/1879614)

[5] Dauphin YN, Pascanu R, Gulcehre C, Cho K, Ganguli S, Bengio Y. 2014 Identifying and attacking the saddle point problem in high-dimensional non-convex optimization. In Advances in neural information processing systems 27 (eds Z Ghahramani, M Welling, C Cortes, ND Lawrence, KQ Weinberger), pp. 2933–2941.

[6] Haut Donahue TL, Hull ML, Rashid MM, Jacobs CR. 2003 How the stiffness of meniscal attachments and meniscal material properties affect tibio-femoral contact pressure computed using a validated finite element model of the human knee joint. J. Biomech. **36**, 19–34. (10.1016/s0021-9290(02)00305-6) 12485635

[7] Donoso-Bravo A, Mailier J, Martin C, Rodríguez J, Aceves-Lara CA, Vande Wouwer A. 2011 Model selection, identification and validation in anaerobic digestion: a review. Water Res. **45**, 5347–5364. (10.1016/j.watres.2011.08.059) 21920578

[8] Donoso-Bravo A, Mailier J, Ruiz-Filippi G, Vande Wouwer A. 2013 Identification in an anaerobic batch system: global sensitivity analysis, multi-start strategy and optimization criterion selection. Bioprocess. Biosyst. Eng. **36**, 35–43. (10.1007/s00449-012-0758-5) 22653035

[9] Ogden RW, Saccomandi G, Sgura I. 2004 Fitting hyperelastic models to experimental data. Comput. Mech. **34**, 484–502. (10.1007/s00466-004-0593-y)

[10] Schmid H, Nash MP, Young AA, Hunter PJ. 2006 Myocardial material parameter estimation—a comparative study for simple shear. J. Biomech. Eng. **128**, 742–750. (10.1115/1.2244576) 16995761

[11] Zhou M, Werbner B, O’Connell G. 2020 Historical review of combined experimental and computational approaches for investigating annulus fibrosus mechanics. J. Biomech. Eng. **142**, 030802. (10.1115/1.4046186) 32005986

[12] Sweigart MA, Zhu CF, Burt DM, DeHoll PD, Agrawal CM, Clanton TO, Athanasiou KA. 2004 Intraspecies and interspecies comparison of the compressive properties of the medial meniscus. Ann. Biomed. Eng. **32**, 1569–1579. (10.1114/b:abme.0000049040.70767.5c) 15636116

[13] Anderson AE, Ellis BJ, Weiss JA. 2007 Verification, validation and sensitivity studies in computational biomechanics. Comput. Methods Biomech. Biomed. Engin. **10**, 171–184. (10.1080/10255840601160484) 17558646 PMC3361760

[14] Avazmohammadi R, Li DS, Leahy T, Shih E, Soares JS, Gorman JH, Gorman RC, Sacks MS. 2018 An integrated inverse model-experimental approach to determine soft tissue three-dimensional constitutive parameters: application to post-infarcted myocardium. Biomech. Model. Mechanobiol. **17**, 31–53. (10.1007/s10237-017-0943-1) 28861630 PMC5809201

[15] Wale ME, Nesbitt DQ, Henderson BS, Fitzpatrick CK, Creechley JJ, Lujan TJ. 2021 Applying ASTM standards to tensile tests of musculoskeletal soft tissue: methods to reduce grip failures and promote reproducibility. J. Biomech. Eng. **143**, 011011. (10.1115/1.4048646) 33006367 PMC7976171

[16] Buschmann MD, Soulhat J, Shirazi-Adl A, Jurvelin JS, Hunziker EB. 1998 Confined compression of articular cartilage: linearity in ramp and sinusoidal tests and the importance of interdigitation and incomplete confinement. J. Biomech. **31**, 171–178. (10.1016/s0021-9290(97)00124-3) 9593212

[17] Bellman R, Åström KJ. 1970 On structural identifiability. Math. Biosci. **7**, 329–339. (10.1016/0025-5564(70)90132-X)

[18] Villaverde AF. 2019 Observability and structural identifiability of nonlinear biological systems. Complexity **2019**, e8497093. (10.1155/2019/8497093)

[19] Walter E, Pronzato L. 1997 Identification of parametric models: from experimental data. New York, NY: Springer.

[20] Chatzis MN, Chatzi EN, Smyth AW. 2015 On the observability and identifiability of nonlinear structural and mechanical systems. Struct. Control Health Monit. **22**, 574–593. (10.1002/stc.1690)

[21] Guillaume JHA *et al*. 2019 Introductory overview of identifiability analysis: a guide to evaluating whether you have the right type of data for your modeling purpose. Environ. Model. Softw. **119**, 418–432. (10.1016/j.envsoft.2019.07.007)

[22] Manski CF. 1995 Identification problems in the social sciences. Cambridge, MA: Harvard University Press.

[23] Miao H, Xia X, Perelson AS, Wu H. 2011 On identifiability of nonlinear ODE models and applications in viral dynamics. SIAM Rev. Soc. Ind. Appl. Math. **53**, 3–39. (10.1137/090757009) 21785515 PMC3140286

[24] Rey Barreiro X, Villaverde AF. 2023 Benchmarking tools for a priori identifiability analysis. Bioinformatics **39**, btad065. (10.1093/bioinformatics/btad065) 36721336 PMC9913045

[25] Massonis G, Banga JR, Villaverde AF. 2021 Structural identifiability and observability of compartmental models of the COVID-19 pandemic. Annu. Rev. Control **51**, 441–459. (10.1016/j.arcontrol.2020.12.001) 33362427 PMC7752088

[26] Transtrum MK, Machta BB, Brown KS, Daniels BC, Myers CR, Sethna JP. 2015 Perspective: sloppiness and emergent theories in physics, biology, and beyond. J. Chem. Phys. **143**, 010901. (10.1063/1.4923066) 26156455

[27] Rothenberg TJ. 1971 Identification in parametric models. Econometrica **39**, 577. (10.2307/1913267)

[28] Koopmans TC, Reiersol O. 1950 The identification of structural characteristics. Ann. Math. Statist. **21**, 165–181. (10.1214/aoms/1177729837)

[29] Read MN, Alden K, Timmis J, Andrews PS. 2020 Strategies for calibrating models of biology. Brief. Bioinf. **21**, 24–35. (10.1093/bib/bby092) 30239570

[30] Hartmann S, Gilbert RR. 2018 Identifiability of material parameters in solid mechanics. Arch. Appl. Mech. **88**, 3–26. (10.1007/s00419-017-1259-4)

[31] Oddes Z, Solav D. 2023 Identifiability of soft tissue constitutive parameters from in-vivo macro-indentation. J. Mech. Behav. Biomed. Mater. **140**, 105708. (10.1016/j.jmbbm.2023.105708) 36801779

[32] Akintunde AR, Miller KS, Schiavazzi DE. 2019 Bayesian inference of constitutive model parameters from uncertain uniaxial experiments on murine tendons. J. Mech. Behav. Biomed. Mater. **96**, 285–300. (10.1016/j.jmbbm.2019.04.037) 31078970 PMC6561498

[33] Fossard AJ, Normand-Cyrot D. 1995 Nonlinear systems: modeling and estimation. London, UK: Chapman & Hall.

[34] Newman HR, DeLucca JF, Peloquin JM, Vresilovic EJ, Elliott DM. 2021 Multiaxial validation of a finite element model of the intervertebral disc with multigenerational fibers to establish residual strain. JOR Spine **4**, e1145. (10.1002/jsp2.1145) 34337333 PMC8313175

[35] Cortes DH, Jacobs NT, DeLucca JF, Elliott DM. 2014 Elastic, permeability and swelling properties of human intervertebral disc tissues: a benchmark for tissue engineering. J. Biomech. **47**, 2088–2094. (10.1016/j.jbiomech.2013.12.021) 24438768 PMC4047194

[36] Yang B, O’Connell GD. 2019 Intervertebral disc swelling maintains strain homeostasis throughout the annulus fibrosus: a finite element analysis of healthy and degenerated discs. Acta Biomater. **100**, 61–74. (10.1016/j.actbio.2019.09.035) 31568880

[37] Holmes MH, Mow VC. 1990 The nonlinear characteristics of soft gels and hydrated connective tissues in ultrafiltration. J. Biomech. **23**, 1145–1156. (10.1016/0021-9290(90)90007-p) 2277049

[38] Upton ML, Guilak F, Laursen TA, Setton LA. 2006 Finite element modeling predictions of region-specific cell-matrix mechanics in the meniscus. Biomech. Model. Mechanobiol. **5**, 140–149. (10.1007/s10237-006-0031-4) 16520958

[39] Danso EK, Mäkelä JTA, Tanska P, Mononen ME, Honkanen JTJ, Jurvelin JS, Töyräs J, Julkunen P, Korhonen RK. 2015 Characterization of site-specific biomechanical properties of human meniscus—importance of collagen and fluid on mechanical nonlinearities. J. Biomech. **48**, 1499–1507. (10.1016/j.jbiomech.2015.01.048) 25708321

[40] Ruiz C, Noailly J, Lacroix D. 2013 Material property discontinuities in intervertebral disc porohyperelastic finite element models generate numerical instabilities due to volumetric strain variations. J. Mech. Behav. Biomed. Mater. **26**, 1–10. (10.1016/j.jmbbm.2013.05.012) 23796430

[41] Galbusera F, Schmidt H, Neidlinger-Wilke C, Wilke HJ. 2011 The effect of degenerative morphological changes of the intervertebral disc on the lumbar spine biomechanics: a poroelastic finite element investigation. Comput. Methods Biomech. Biomed. Engin. **14**, 729–739. (10.1080/10255842.2010.493522) 21390934

[42] Schmidt H, Bashkuev M, Galbusera F, Wilke HJ, Shirazi-Adl A. 2014 Finite element study of human lumbar disc nucleus replacements. Comput. Methods Biomech. Biomed. Engin. **17**, 1762–1776. (10.1080/10255842.2013.766722) 23477684

[43] Wilson W, van Donkelaar CC, van Rietbergen B, Huiskes R. 2005 A fibril-reinforced poroviscoelastic swelling model for articular cartilage. J. Biomech. **38**, 1195–1204. (10.1016/j.jbiomech.2004.07.003) 15863103

[44] Moore AC, DeLucca JF, Elliott DM, Burris DL. 2016 Quantifying cartilage contact modulus, tension modulus, and permeability with Hertzian biphasic creep. J. Tribol. **138**, 0414051–0414057. (10.1115/1.4032917) 27536012 PMC4967882

[45] Zimmerman BK, Nims RJ, Chen A, Hung CT, Ateshian GA. 2021 Direct osmotic pressure measurements in articular cartilage demonstrate nonideal and concentration-dependent phenomena. J. Biomech. Eng. **143**, 041007. (10.1115/1.4049158) 33210125 PMC7872001

[46] Macirowski T, Tepic S, Mann RW. 1994 Cartilage stresses in the human hip joint. J. Biomech. Eng. **116**, 10–18. (10.1115/1.2895693) 8189704

[47] Ateshian GA, Lai WM, Zhu WB, Mow VC. 1994 An asymptotic solution for the contact of two biphasic cartilage layers. J. Biomech. **27**, 1347–1360. (10.1016/0021-9290(94)90044-2) 7798285

[48] Ateshian GA, Wang H. 1995 A theoretical solution for the frictionless rolling contact of cylindrical biphasic articular cartilage layers. J. Biomech. **28**, 1341–1355. (10.1016/0021-9290(95)00008-6) 8522547

[49] Park S, Krishnan R, Nicoll SB, Ateshian GA. 2003 Cartilage interstitial fluid load support in unconfined compression. J. Biomech. **36**, 1785–1796. (10.1016/s0021-9290(03)00231-8) 14614932 PMC2833094

[50] Zimmerman BK, Bonnevie ED, Park M, Zhou Y, Wang L, Burris DL, Lu XL. 2015 Role of interstitial fluid pressurization in TMJ lubrication. J. Dent. Res. **94**, 85–92. (10.1177/0022034514553626) 25297115 PMC4270807

[51] Mow VC, Gu WY, Chen FH. 2005 Structure and function of articular cartilage and meniscus. In Basic orthopaedic biomechanics & mechano-biology, pp. 181–258. Philadelphia, PA: Lippincott Williams & Wilkins.

[52] Ateshian GA, Warden WH, Kim JJ, Grelsamer RP, Mow VC. 1997 Finite deformation biphasic material properties of bovine articular cartilage from confined compression experiments. J. Biomech. **30**, 1157–1164. (10.1016/s0021-9290(97)85606-0) 9456384

[53] Mow VC, Kuei SC, Lai WM, Armstrong CG. 1980 Biphasic creep and stress relaxation of articular cartilage in compression: theory and experiments. J. Biomech. Eng. **102**, 73–84. (10.1115/1.3138202) 7382457

[54] Athanasiou KA, Rosenwasser MP, Buckwalter JA, Malinin TI, Mow VC. 1991 Interspecies comparisons of in situ intrinsic mechanical properties of distal femoral cartilage. J. Orthop. Res. **9**, 330–340. (10.1002/jor.1100090304) 2010837

[55] Athanasiou KA, Agarwal A, Muffoletto A, Dzida FJ, Constantinides G, Clem M. 1995 Biomechanical properties of hip cartilage in experimental animal models. Clin. Orthop. Relat. Res. **1995**, 254–266. 7634715

[56] Froimson MI, Ratcliffe A, Gardner TR, Mow VC. 1997 Differences in patellofemoral joint cartilage material properties and their significance to the etiology of cartilage surface fibrillation. Osteoarthr. Cartil. **5**, 377–386. (10.1016/S1063-4584(97)80042-8) 9536286

[57] Mow VC, Gibbs MC, Lai WM, Zhu WB, Athanasiou KA. 1989 Biphasic indentation of articular cartilage—II. A numerical algorithm and an experimental study. J. Biomech. **22**, 853–861. (10.1016/0021-9290(89)90069-9) 2613721

[58] Safa BN, Santare MH, Ethier CR, Elliott DM. 2021 Identifiability of tissue material parameters from uniaxial tests using multi-start optimization. Acta Biomater. **123**, 197–207. (10.1016/j.actbio.2021.01.006) 33444797 PMC8518191

[59] Eskandari M, Nordgren TM, O’Connell GD. 2019 Mechanics of pulmonary airways: linking structure to function through constitutive modeling, biochemistry, and histology. Acta Biomater. **97**, 513–523. (10.1016/j.actbio.2019.07.020) 31330329 PMC7462120

[60] Peloquin JM, Santare MH, Elliott DM. 2016 Advances in quantification of meniscus tensile mechanics including nonlinearity, yield, and failure. J. Biomech. Eng. **138**, 021002. (10.1115/1.4032354) 26720401 PMC4844065

[61] Szczesny SE, Peloquin JM, Cortes DH, Kadlowec JA, Soslowsky LJ, Elliott DM. 2012 Biaxial tensile testing and constitutive modeling of human supraspinatus tendon. J. Biomech. Eng. **134**, 021004. (10.1115/1.4005852) 22482671 PMC3646567

[62] Maas SA, Ellis BJ, Ateshian GA, Weiss JA. 2012 FEBio: finite elements for biomechanics. J. Biomech. Eng. **134**, 011005. (10.1115/1.4005694) 22482660 PMC3705975

[63] Patel JM, Wise BC, Bonnevie ED, Mauck RL. 2019 A systematic review and guide to mechanical testing for articular cartilage tissue engineering. Tissue Eng. Part C. Methods **25**, 593–608. (10.1089/ten.TEC.2019.0116) 31288616 PMC6791482

[64] Klika V, Gaffney EA, Chen YC, Brown CP. 2016 An overview of multiphase cartilage mechanical modelling and its role in understanding function and pathology. J. Mech. Behav. Biomed. Mater. **62**, 139–157. (10.1016/j.jmbbm.2016.04.032) 27195911

[65] Taylor ZA, Miller K. 2006 Constitutive modeling of cartilaginous tissues: a review. J. Appl. Biomech. **22**, 212–229. (10.1123/jab.22.3.212) 17215553

[66] Marchiori G, Berni M, Boi M, Filardo G. 2019 Cartilage mechanical tests: evolution of current standards for cartilage repair and tissue engineering. A literature review. Clin Biomech **68**, 58–72. (10.1016/j.clinbiomech.2019.05.019) 31158591

[67] Gu WY, Yao H, Huang CY, Cheung HS. 2003 New insight into deformation-dependent hydraulic permeability of gels and cartilage, and dynamic behavior of agarose gels in confined compression. J. Biomech. **36**, 593–598. (10.1016/s0021-9290(02)00437-2) 12600349

[68] Korhonen RK, Laasanen MS, Töyräs J, Rieppo J, Hirvonen J, Helminen HJ, Jurvelin JS. 2002 Comparison of the equilibrium response of articular cartilage in unconfined compression, confined compression and indentation. J. Biomech. **35**, 903–909. (10.1016/s0021-9290(02)00052-0) 12052392

[69] Jurvelin JS, Buschmann MD, Hunziker EB. 2003 Mechanical anisotropy of the human knee articular cartilage in compression. Proc. Inst. Mech. Eng. H **217**, 215–219. (10.1243/095441103765212712) 12807162

[70] Römgens AM, van Donkelaar CC, Ito K. 2013 Contribution of collagen fibers to the compressive stiffness of cartilaginous tissues. Biomech. Model. Mechanobiol. **12**, 1221–1231. (10.1007/s10237-013-0477-0) 23443749

[71] DeLucca JF, Cortes DH, Jacobs NT, Vresilovic EJ, Duncan RL, Elliott DM. 2016 Human cartilage endplate permeability varies with degeneration and intervertebral disc site. J. Biomech. **49**, 550–557. (10.1016/j.jbiomech.2016.01.007) 26874969 PMC4779374

[72] Iatridis JC, Setton LA, Foster RJ, Rawlins BA, Weidenbaum M, Mow VC. 1998 Degeneration affects the anisotropic and nonlinear behaviors of human anulus fibrosus in compression. J. Biomech. **31**, 535–544. (10.1016/s0021-9290(98)00046-3) 9755038

[73] Wu Y, Cisewski SE, Sun Y, Damon BJ, Sachs BL, Pellegrini VD, Slate EH, Yao H. 2017 Quantifying baseline fixed charge density in healthy human cartilage endplate: a two-point electrical conductivity method. Spine **42**, E1002–E1009. (10.1097/BRS.0000000000002061) 28699925 PMC5509527

[74] Athanasiou KA, Natoli RM. 2009 Biphasic theory. In Introduction to continuum biomechanics, pp. 179–198. Synthesis Lectures on Biomedical Engineering. Cham, Switzerland: Springer International Publishing. (10.1007/978-3-031-01626_49)

[75] Ateshian GA. 2017 Mixture theory for modeling biological tissues: illustrations from articular cartilage. In Biomechanics: trends in modeling and simulation (eds GA Holzapfel, RW Ogden), pp. 1–51. Cham, Switzerland: Springer International Publishing. (10.1007/978-3-319-41411)

[76] Jacobs NT, Cortes DH, Peloquin JM, Vresilovic EJ, Elliott DM. 2014 Validation and application of an intervertebral disc finite element model utilizing independently constructed tissue-level constitutive formulations that are nonlinear, anisotropic, and time-dependent. J. Biomech. **47**, 2540–2546. (10.1016/j.jbiomech.2014.06.008) 24998992 PMC4366133

[77] Cortes DH, Elliott DM. 2012 Extra-fibrillar matrix mechanics of annulus fibrosus in tension and compression. Biomech. Model. Mechanobiol. **11**, 781–790. (10.1007/s10237-011-0351-x) 21964839 PMC3500513

[78] Maas SA, Erdemir A, Halloran JP, Weiss JA. 2016 A general framework for application of prestrain to computational models of biological materials. J. Mech. Behav. Biomed. Mater. **61**, 499–510. (10.1016/j.jmbbm.2016.04.012) 27131609 PMC7651410

[79] Yang B, O’Connell GD. 2019 GAG content, fiber stiffness, and fiber angle affect swelling-based residual stress in the intact annulus fibrosus. Biomech. Model. Mechanobiol. **18**, 617–630. (10.1007/s10237-018-1105-9) 30535612

[80] Cortes DH, Han WM, Smith LJ, Elliott DM. 2013 Mechanical properties of the extra-fibrillar matrix of human annulus fibrosus are location and age dependent. J. Orthop. Res. **31**, 1725–1732. (10.1002/jor.22430) 23818058 PMC4164199

[81] Périé D, Korda D, Iatridis JC. 2005 Confined compression experiments on bovine nucleus pulposus and annulus fibrosus: sensitivity of the experiment in the determination of compressive modulus and hydraulic permeability. J. Biomech. **38**, 2164–2171. (10.1016/j.jbiomech.2004.10.002) 16154403

[82] Mannakee BK, Ragsdale AP, Transtrum MK, Gutenkunst RN. 2016 Sloppiness and the geometry of parameter space. In Uncertainty in biology: a computational modeling approach (eds L Geris, D Gomez-Cabrero), pp. 271–299. Cham, Switzerland: Springer International Publishing. (10.1007/978-3-319-21296-8_11)

[83] Gutenkunst RN, Waterfall JJ, Casey FP, Brown KS, Myers CR, Sethna JP. 2007 Universally sloppy parameter sensitivities in systems biology. PLoS Comput. Biol. **3**, 1871–1878. (10.1371/journal.pcbi.0030189) 17922568 PMC2000971

[84] Apgar JF, Witmer DK, White FM, Tidor B. 2010 Sloppy models, parameter uncertainty, and the role of experimental design. Mol. Biosyst. **6**, 1890–1900. (10.1039/b918098b) 20556289 PMC3505121

[85] Richard Shewchuk J. 1997 Adaptive precision floating-point arithmetic and fast robust geometric predicates. Discrete Comput. Geom. **18**, 305–363. (10.1007/PL00009321)

[86] Goodsell MD, Joury A. 2022 Active learning BSM parameter spaces. Eur. Phys. J. C. **83**. (10.1140/epjc/s10052-023-11368-3)

[87] Chambers JM. 1973 Fitting nonlinear models: numerical techniques. Biometrika **60**, 1–13. (10.1093/biomet/60.1.1)

[88] Zhang Y, Van Bael A, Andrade-Campos A, Coppieters S. 2022 Parameter identifiability analysis: mitigating the non-uniqueness issue in the inverse identification of an anisotropic yield function. Int. J. Solids Struct. **243**, 111543. (10.1016/j.ijsolstr.2022.111543)

[89] Mathur R. 2012 An analytical approach to computing step sizes for finite-difference derivatives. Austin, TX: The University of Texas. See https://repositories.lib.utexas.edu/handle/2152/ETD-UT-2012-05-5275.

[90] Iott J, Haftka R, Adelman HM. 1985 Selecting step sizes in sensitivity analysis by finite differences. National Aeronautics and Space Administration. See https://ntrs.nasa.gov/citations/19850025225.

[91] Eisen MB, Spellman PT, Brown PO, Botstein D. 1998 Cluster analysis and display of genome-wide expression patterns. Proc. Natl Acad. Sci. USA **95**, 14 863–14 868. (10.1073/pnas.95.25.14863) PMC24541 9843981

[92] Ramoni MF, Sebastiani P, Kohane IS. 2002 Cluster analysis of gene expression dynamics. Proc. Natl Acad. Sci. USA **99**, 9121–9126. (10.1073/pnas.132656399) 12082179 PMC123104

[93] Gill J, King G. 2004 Numerical issues involved in inverting Hessian matrices. In Numerical issues in statistical computing for the social scientist, pp. 143–176. Hoboken, NJ: John Wiley & Sons, Inc. (10.1002/0471475769)

[94] Davidson R, MacKinnon JG. 2003 Econometric theory and methods. New York, NY: Oxford University Press.

[95] Holmes MH, Lai WM, Mow VC. 1985 Singular perturbation analysis of the nonlinear, flow-dependent compressive stress relaxation behavior of articular cartilage. J. Biomech. Eng. **107**, 206–218. (10.1115/1.3138545) 4046561

[96] Lai WM, Mow VC. 1980 Drag-induced compression of articular cartilage during a permeation experiment. Biorheology **17**, 111–123. (10.3233/bir-1980-171-213) 7407341

[97] Zeng K, Breder K, Rowcliffe DJ, Herrström C. 1992 Elastic modulus determined by Hertzian indentation. J. Mater. Sci. **27**, 3789–3792. (10.1007/BF00545457)

[98] Lu XL, Sun DDN, Guo XE, Chen FH, Lai WM, Mow VC. 2004 Indentation determined mechanoelectrochemical properties and fixed charge density of articular cartilage. Ann. Biomed. Eng. **32**, 370–379. (10.1023/b:abme.0000017534.06921.24) 15095811

[99] Oyen ML. 2008 Poroelastic nanoindentation responses of hydrated bone. J. Mater. Res. **23**, 1307–1314. (10.1557/JMR.2008.0156)

[100] Boschetti F, Pennati G, Gervaso F, Peretti GM, Dubini G. 2004 Biomechanical properties of human articular cartilage under compressive loads. Biorheology **41**, 159–166. 15299249

[101] Miller GJ, Morgan EF. 2010 Use of microindentation to characterize the mechanical properties of articular cartilage: comparison of biphasic material properties across length scales. Osteoarthr. Cartil. **18**, 1051–1057. (10.1016/j.joca.2010.04.007) PMC2906658 20417292

[102] Soltz MA, Ateshian GA. 2000 A conewise linear elasticity mixture model for the analysis of tension-compression nonlinearity in articular cartilage. J. Biomech. Eng. **122**, 576–586. (10.1115/1.1324669) 11192377 PMC2854000

[103] Wagner PR, Nagel J, Sudret B. 2022 UQLab user manual – Bayesian inference for model calibration and inverse problems. Report UQLab-V2.0-113. Zurich, Switzerland: Chair of Risk, Safety and Uncertainty Quantification.

[104] Salvador M, Regazzoni F, Dede’ L, Quarteroni A. 2023 Fast and robust parameter estimation with uncertainty quantification for the cardiac function. Comput. Methods Programs Biomed. **231**, 107402. (10.1016/j.cmpb.2023.107402) 36773593

[105] Matott LS, Babendreier JE, Purucker ST. 2009 Evaluating uncertainty in integrated environmental models: a review of concepts and tools. Water Resour. Res. **45**. (10.1029/2008WR007301)

[106] Gustafson P. 2005 On model expansion, model contraction, identifiability and prior information: two illustrative scenarios involving mismeasured variables. Stat. Sci. **20**, 111–140. (10.1214/088342305000000098)

[107] Narayan A *et al*. 2023 UncertainSCI: uncertainty quantification for computational models in biomedicine and bioengineering. Comput. Biol. Med. **152**, 106407. (10.1016/j.compbiomed.2022.106407) 36521358 PMC9812870

[108] Tennøe S, Halnes G, Einevoll GT. 2018 Uncertainpy: a Python toolbox for uncertainty quantification and sensitivity analysis in computational neuroscience. Front. Neuroinform. **12**, 49. (10.3389/fninf.2018.00049) 30154710 PMC6102374

[109] Seo J, Schiavazzi DE, Kahn AM, Marsden AL. 2020 The effects of clinically-derived parametric data uncertainty in patient-specific coronary simulations with deformable walls. Int. J. Numer. Method. Biomed. Eng. **36**, e3351. (10.1002/cnm.3351) 32419369 PMC8211426

[110] Raue A, Kreutz C, Maiwald T, Bachmann J, Schilling M, Klingmüller U, Timmer J. 2009 Structural and practical identifiability analysis of partially observed dynamical models by exploiting the profile likelihood. Bioinformatics **25**, 1923–1929. (10.1093/bioinformatics/btp358) 19505944

[111] Cherkassky V, Mulier FM. 2007 Learning from data: concepts, theory, and methods, 2nd edn. Hoboken, NJ: Wiley-IEEE Press.

[112] Peloquin JM, Elliott D. 2024 Data from: Global and local identifiability analysis of a nonlinear biphasic constitutive model in confined compression. Figshare. (10.6084/m9.figshare.c.7492732) PMC11557236 39532129

